# Construction and application of a *Xanthomonas campestris* CGMCC15155 strain that produces white xanthan gum

**DOI:** 10.1002/mbo3.631

**Published:** 2018-04-15

**Authors:** Xiaohui Dai, Ge Gao, Mengmeng Wu, Weiying Wei, Jianmei Qu, Guoqiang Li, Ting Ma

**Affiliations:** ^1^ Key Laboratory of Molecular Microbiology Technology Ministry of Education College of Life Sciences Nankai University Tianjin China; ^2^ Tianjin Engineering Technology Center of Green Manufacturing Biobased Materials Tianjin China

**Keywords:** VHb, Xanthan gum, Xanthomonadins, *Xanthomonas campestris*

## Abstract

In the industrial production of xanthan gum using *Xanthomonas campestris* CGMCC15155, large amounts of ethanol are required to extract xanthan gum from the fermentation broth and remove xanthomonadin impurities. To reduce the amount of ethanol and the overall production cost of xanthan gum, a xanthomonadin‐deficient strain of CGMCC15155 was constructed by inserting the *Vitreoscilla* globin (*vgb*) gene, under the control of the LacZ promoter, into the region of the *pigA* gene, which is involved in xanthomonadin synthesis. The insertion of *vgb* inactivated *pigA*, resulting in the production of white xanthan gum. The lack of xanthomonadins resulted in a decreased yield of xanthan gum. However, the expression product of *vgb* gene, VHb, could increase the metabolism of *X. campestris*, which allowed the production of xanthan gum to reach wild‐type levels in the engineered strain. The yield, molecular weight, and rheological properties of the xanthan gum synthesized by the engineered and wild‐type bacteria were essentially the same. When the same volume of ethanol was used, the whiteness values of the xanthan gum extracted from engineered and wild‐type bacteria were 65.20 and 38.17, respectively. To extract xanthan gum with the same whiteness, three and seven times the fermentation volume of ethanol was required for the engineered and wild‐type strains, respectively. Thus, the engineered train reduced the requirement for ethanol in xanthan gum production by 133.3%. The results demonstrated that the engineered bacteria used less ethanol, thus reducing the downstream processing cost in xanthan gum production.

## 1 INTRODUCTION

Polysaccharides are very important biological materials in food, medicine, and other industries. Microbial polysaccharides have many advantages, such as taking much less time to prepare polysaccharides using a bioreactor than extracting them from plants; and they can be produced using industrial raw materials or even waste materials as carbon sources (Donot, Fontana, Baccou, & Schorr‐Galindo, 2012; Kojić, Vrvić, Gojgić‐Cvijović, Beškoski, & Jakovljević, 2016). However, the polysaccharides from algae and higher plant plants dominate the world polysaccharide market, while polysaccharides derived from microorganisms are still not fully utilized, mainly because, in some cases, the production of polysaccharides by microorganisms demands strict requirements of the bioreactor conditions, leading to high production costs. Only a few microbial polysaccharides with important commercial value, such as bacterial cellulose, which can be used as biomaterials, or xanthan and gellan gums, which change the rheological properties of aqueous solutions, have been widely applied in industry (Freitas, Alves, & Reis, 2011). Recent research has focused on understanding the pathway of polysaccharide biosynthesis and improving the productivity of various extracellular polymeric substances (EPSs) via genetic engineering and metabolic engineering to reduce costs. Most of these studies aimed to increase the sugar nucleotide pool (EPS precursors) to enhance the carbon flux of the final polymer (Huang et al., 2013; Schmid, Sieber, & Rehm, 2015) or to overexpress the genes involved in EPS assembly (Jones, 2012; Pollock, Yamazaki, Thorne, Mikolajczak, & Armentrout, 1998). In addition, disruption of pathways that compete with EPS precursor synthesis could also increase productivity (Galindo, Peña, Núñez, Segura, & Espín, 2007; Pena et al., 2002).

Xanthan gum is a neutral, water‐soluble polysaccharide that is produced by *Xanthomonas campestris* (Northern Regional Research Laboratories B‐1459) and was discovered in the 1950s (Margaritis & Zajic, 1978). Low‐concentration solutions of xanthan gum are highly viscous and have unique pseudoplastic rheological properties. Xanthan gum also exhibits high shear stability and is insensitive to temperature, pH, and salt concentration. For these reasons, xanthan gum is a critical product in the microbial gum market and is used in a variety of industries, including medicine, petroleum, and cosmetics (Sandvik & Maerker, 1977). Xanthan gum is employed in the petroleum industry because of its pseudoplastic rheological properties; the application of low‐concentration xanthan gum solutions can maintain the viscosity and control the rheological properties of the drilling fluid. In the food industry, xanthan gum is used primarily as a molding agent, stabilizer, and thickener. The addition of small amounts of xanthan gum to beverages can enhance their taste and prevent insoluble ingredients in juice‐type beverages form precipitating. The United States Food and Drug Administration approved xanthan gum as a food additive in 1969, and European countries have also approved its use in the food industry.

Xanthan products are divided into four grades: crude grade, industrial grade, food grade, and medicinal auxiliary grade. Among them, food grade and medicinal auxiliary grade xanthan gum have the highest quality, purity, and associated production costs. The production cost of food grade xanthan gum in the downstream purification steps can be as high as 50%, which would not be necessary for nonfood applications (Palaniraj & Jayaraman, 2011). During the production of xanthan gum, *Xanthomonas* produces xanthomonadins, a class of water‐insoluble, brominated, aryl polyene yellow pigments (Amanullah, Tuttiett, & Nienow, 1998). Xanthomonadins play important roles in the survival and pathogenesis of *Xanthomonas* by protecting the bacteria from photobiological damage; providing antioxidant activity; promoting their ability to infect cruciferous plants, leading to black rot (Goel, Rajagopal, & Sonti, 2001); and aiding the synthesis extracellular polysaccharides. Xanthomonadins attach to the outer cell membranes of *Xanthomonas* cells (Stephens & Starr, 1963); thus, xanthan gum contains xanthomonadins as pigment impurities, which cause difficulties in the purification process. In industrial production, ethanol is usually used to precipitate xanthan gum (Garcıa‐Ochoa, Santos, Casas, & Gomez, 2000; Zhang & Chen, 2010). The xanthomonadins dissolve in organic reagents (Nasr, Soudi, & Haghighi, 2007). However, the fermentation broth of xanthan gum is highly viscous and contains many impurities, making its purification difficult. When using ethanol at three times the volume of the fermentation broth to extract xanthan gum, some of the pigment remains and affects the quality of the xanthan gum such that it does not meet the application standards of the food industry. Therefore, more ethanol is needed to remove the pigments. Wu et al., (2011) obtained a carotenoid‐free *Sphingomonas paucimobilis* mutant strain by chemical and UV mutagenesis for the commercial production of gellan‐gum, and Zhang et al. (2016) obtained a white welan gum producing strain by knocking out genes related to carotenoid synthesis in *Alcaligenes* sp. Thus, constructing strains defective in xanthomonadin production could reduce the consumption of ethanol and reduce the production cost of xanthan gum.

Despite its wide application, some problems in xanthan gum production persist. *X. campestris* is a strictly aerobic bacterium, and the rate of oxygen transmission affects xanthan gum production (Becker, Katzen, Pühler, & Ielpi, 1998). One of the main problems in industrial xanthan gum production is the difficulty in stirring and ventilating the broth during fermentation, which affects oxygen transmission (Palaniraj & Jayaraman, 2011). Poor oxygen transmission reduces the molecular weight of the produced xanthan gum (Suh, Herbst, Schumpe, & Deckwer, 1990). *Vitreoscilla* hemoglobin (VHb), which is produced by the gram‐negative bacterium *Vitreoscilla*, is the only hemoglobin found in prokaryotes (Tyree & Webster, 1978). VHb is a homodimer with a molecular weight of 32,783 Da and can exist in three states (oxidized, reduced, and oxygenated) depending on the environmental conditions. The oxygenated state is stable in oxygen‐enriched conditions, while the reduced state is the physiologically active state (Zhang et al., 2007). The expression of VHb has been shown to promote oxygen diffusion and metabolism in the host; however, the mechanism of action of VHb in cells remains unclear; different mechanisms related to diffusion promotion, the redox effect, and terminal electron acceptors have been proposed (Frey, 2002). The heterologous expression of VHb promotes cell growth and metabolite synthesis (Kang, Kim, & Cha, 2002; Khosla & Bailey, 1988; Zhang et al., 2013), thereby overcoming the aerobic limits in the fermentation process and supporting the aerobic growth of bacteria under oxygen‐poor conditions (Wakabayashi, Matsubara, & Webster, 1986). Thus, we hypothesized that introducing the *Vitreoscilla* globin (*vgb*) gene into *X. campestris* might reduce the oxygen shortage, resulting from the excessive viscosity of the fermentation broth, thereby increasing the yield of xanthan gum.

In this study, a genetically engineered strain of *X. campestris* CGMCC15155 was constructed by inserting the *vgb* gene, under the control of the LacZ promoter, into the *pigA* gene, which is a key gene in xanthomonadin synthesis. The lack of xanthomonadins resulted in a decrease in the yield of xanthan gum. However, the expression of VHb increased the metabolism of *X. campestris*, allowing the engineered strain to produce xanthan gum at wild‐type levels. The results demonstrated that the engineered bacteria required less ethanol for downstream processing, thus reducing the cost of xanthan gum production.

## 2 MATERIALS AND METHODS

### 2.1 Strains and culture conditions

The strains and plasmids used are listed in Table 1. *Escherichia coli* S17‐1 was grown in lysogeny broth (10 g peptone, 5 g yeast extract, and 5 g sodium chloride per liter) at 37°C. The composition of the seed medium for *X campestris* CGMCC15155 was as follows (g/L): 20 g of sucrose; 3 g of peptone; 1 g of yeast extract; and 5 g beef extract (pH 7.0 ± 0.02). The composition of the fermentation medium was (g/L): 40 g of sucrose; 4 g of soy peptone; and 4 g of calcium carbonate.

**Table 1:** Strains and plasmids

Strains and plasmids	Relevant genotype and characteristics	References
Strains		
* Escherichia. coli* S17‐1	recA pro hsdR RP4‐2‐Tc::Mu‐Km::Tn7	Mazodier, Petter, and Thompson (1989)
* Xanthomonas campestris*	Wild type, Cm^r^	CGMCC15155
* *Δ*pigA*	XC derivative, Δ*pigA*	This work
* *Δ*pigA*::*vgb*	XC derivative, Δ*pigA*, lacZ‐*vgb*	This work
Plasmids		
* *pBBR‐*vgb*	oriT, Km^r^, LacZ‐*vgb*	Zhang et al. (2016)
* *pLO3	sacB, Tc^r^, oriT	Lenz and Friedrich (1998)
* *pLO3‐*pigA*	pLO3 derivation with deletion fragment of *pigA*	This work
* *pLO3‐*vgb*	pLO3 derivation with insertion fragment of PlacZ*‐vgb*	This work

A single colony was inoculated into 5 ml of seed medium and grown for 20 hr with shaking at 30°C. The culture was then inoculated into 100 ml of seed medium as a 1% (v/v) inoculation and then grown for 20 hr with shaking at 30°C. The cells were then inoculated into 500 ml flasks as a 10% (v/v) inoculation and cultivated for 66 hr with shaking at 28°C.

The batch fermentations were performed in a 5 L bioreactor containing 3.2 L of medium. The fermentation medium contained the following (g/L): 40 g of sucrose and 4 g of Soy peptone. The culture temperature was 30°C, and pH was maintained at 7.0 ± 0.02 using 1 mol/L NaOH and 1 mol/L HCl. In the fermentation process, the agitation rate was 400 rpm during the first 24 hr and 600 rpm during the next 42 hr; the aeration rate was 0.4 vvm.

### 2.2 Construction of mutant strains

The oligonucleotide primers used to construct the plasmids are listed in Table S1. We used the suicide vector pLO3 to construct the gene knockout vector pLO3‐*pigA*. We used PrimeSTAR DNA Polymerase (Takara Bio, Tokyo, Japan) and primers *pigA*‐1SF/*pigA*‐1SR and *pigA*‐1XF/*pigA*‐1XR to amplify the upstream and downstream homologous arm sequences of the *pigA* gene. Overlap polymerase chain reaction (PCR) was used to connect the upstream homologous arm and the downstream homologous arm. Restriction endonucleases SacI and XbaI were used to digest the recombinant fragments and the pLO3 plasmid. T4 DNA ligase was then used to ligate the digested DNA fragments and the plasmid. The knockout plasmid pLO3‐*pigA* was obtained and transformed into *E. coli* S17‐1.

We used suicide vector pLO3 to construct the gene insertion vector pLO3‐*vgb*. Primers P*vgb*‐F/P*vgb*‐R were used to amplify the sequence of PlacZ‐*vgb* from pBBR‐*vgb*. Primers *pigA*‐2SF/*pigA*‐2SR and *pigA*‐2XF/*pigA*‐2XR were used to amplify the upstream and downstream homologous arm sequences of the *pigA* gene, respectively. The three DNA fragments were connected using overlap PCR. Subsequently, the recombinant fragments were digested using restriction endonucleases SacI and XbaI and ligated with suicide plasmid pLO3. Insertion vector pLO3‐*vgb* was obtained and transformed into *E. coli* S17‐1.

**Figure 1:**
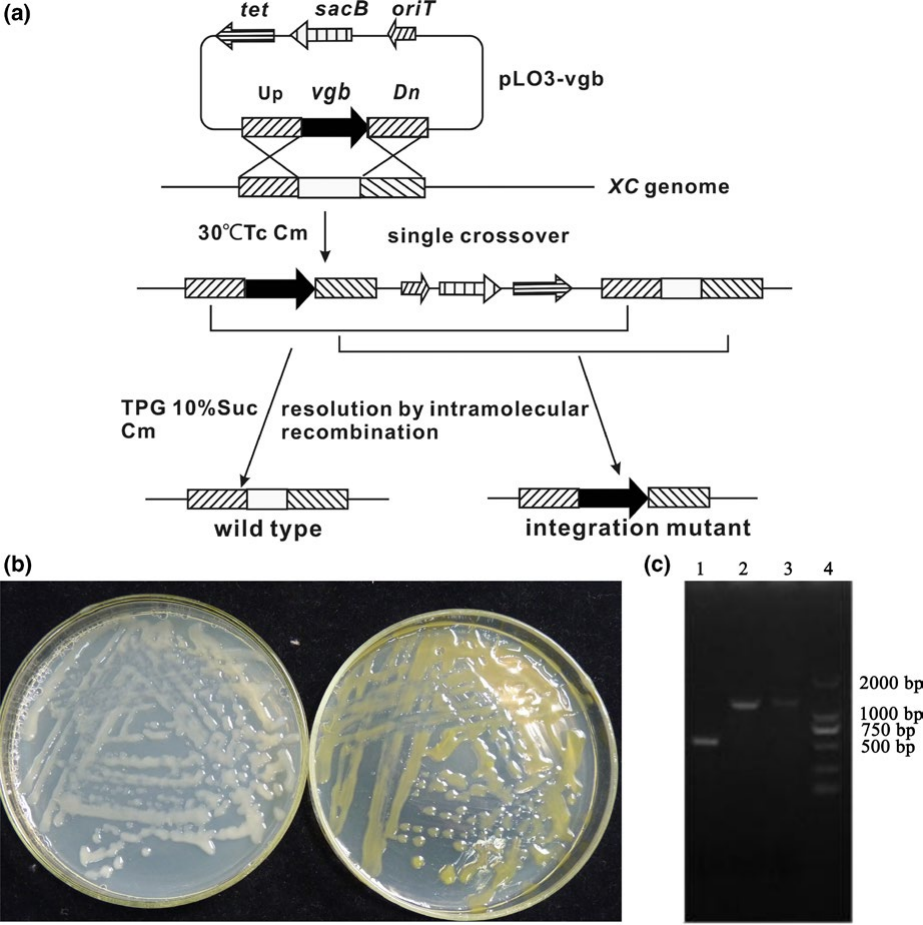
Construction and confirmation of engineered strains. (a) Construction of mutant strains. pLO3‐*vgb* was transferred to *XC* via conjugation, and the single‐exchange recombinants were screened by chloramphenicol and tetracycline double‐resistance plates. The obtained single‐exchange recombinants were screened on TPG plates containing 10% sucrose to obtain the desired mutants, which were verified by PCR. The initial recombination could occur at the upstream or downstream part of the homologous arm; the final chromosomal structure was the same in both cases. (*Tet*, tetracycline resistance protein encoding gene; *sacB*, levansucrase; *Orit*, origin of transfer; Cm, chloromycetin; Up, *pigA* upstream homologous arm; Dn, *pigA* downstream homologous arm; *vgb*,* Vitreoscilla* globin gene; TPG, TPG agarose plate; Suc, sucrose.) (b) Engineered strain Δ*pigA*::*vgb* (left) and wild type (right) incubated for 48 hr on TPG plates at 28°C. (c) Δ*pigA*::*vgb* verification. Lane 1, Δ*pigA*::*vgb*; Lanes 2 and 3, wild type; Lane 4, DL2000 marker. The two strains were tested by PCR using the primer *pigA* 2F/*pigA* 2R, the length of *vgb* is 537 bp, and the length of *pigA* is 1535 bp

The construction of mutant strains based on gene knockout with homologous recombination is shown in Figure 1. Recombinant plasmid pLO3‐*pigA* was transformed into *X. campestris* CGMCC15155 (XC) via conjugation between *E. coli* S17‐1 pLO3‐*pigA* and XC, which resulted in the *pigA* gene being knocked out by homologous recombination. The method to construct strain Δ*pigA*::*vgb* was the same as that mentioned above.

### 2.3 Quantitative real‐time PCR analyses

Wild‐type and Δ*pigA*::*vgb* strains were grown in liquid medium at 30°C for 16 hr with shaking. Total RNA was extracted by using an RNAprep pure Cell/Bacteria Kit (Tiangen, Beijing, China). The RNA concentration and quality were determined using a spectrometer (BioDrop, Cambridge, UK). Total RNA was treated with gDNase to avoid interference of genomic DNA, and the total RNA obtained was reverse transcribed into cDNA using a Quantscript RT Kit (Tiangen). RT‐PCR was performed on a Real‐Time PCR System (Bio‐Rad, CA, USA) with the Bestar^®^ SybGreen QPCR Mastermix (DBI Bioscience, China). The 16s rDNA and *vgb* genes were amplified using primers 16sdl1/16sdl2 and *vgb*dl1/*vgb*dl2 (these primers are shown in Table S1), respectively, and all the reactions were performed in triplicate. Expression of the 16s rDNA was used as an internal standard to normalize the quantifications. The relative expression level of *vgb* was calculated using the 2^−ΔΔCT^ method.

### 2.4 CO differential chromatography

Wild‐type and Δ*pigA*::*vgb* bacteria were grown in liquid culture medium for 16 hr at 30°C with shaking. The cells were then harvested by centrifugation at 8,000*g* for 5 min and washed three times with 0.1 mol/L phosphate buffer. The pellet was resuspended in 4 ml of phosphate buffer and sonicated on ice using a JY92‐II sonicator (SCIENTZ, Ningbo, China). The procedure comprised work 3 s, intermittent 3 s, for a total of 300 s at 300 W. The unbroken cells and cell debris were removed by centrifugation at 12,000*g* for 15 min. Subsequently, excess sodium dithionite mother liquor was added to the supernatant to a final concentration of 2.5 mg/ml. The sample was divided into two equal parts after shaking, and each part was mixed well. One part was bubbled with CO, and the second was bubbled with air. The light absorption values of the samples in the range of 400–500 nm were then measured using a spectrophotometer (Biodrop).

### 2.5 Analytical methods

#### 2.5.1 Determination of xanthan gum

Ethanol (three times the volume of the fermentation broth) was added to the fermentation broth and mixed well. The xanthan gum was then precipitated, filtered, and dried in an oven at 90°C until it reached a constant weight. The xanthan gum yield was calculated according to the xanthan gum quantity per liter of fermentation broth after weighing using a precision balance.

#### 2.5.2 Viscosity measurement

The viscosity of the fermentation broth was measured using a Brinell viscometer LV‐DV_II + (Brookfield, Middleboro, USA) with No. 64 rotor at 60 rpm. Each measurement used 18 ml broth and was repeated three times.

#### 2.5.3 Determination of biomass

Spectrophotometry was used to measure the biomass. Fermentation broth (5 ml) was diluted with 45 ml of deionized water and then centrifuged at 36,000*g* for 30 min to collect the cells. The cells were resuspended in 40 ml of 0.9% (w/w) sodium chloride solution, centrifuged at 36,000*g* for 10 min, and then washed three times. Then, the cells were collected and suspended in 5 ml of deionized water, and their optical density was measured at a wavelength of 600 nm using a spectrophotometer (Biodrop).

#### 2.5.4 Molecular weight determination of xanthan gum

The molecular weight of xanthan gum was determined by gel permeation chromatography (GPC). The xanthan gum was dissolved in ultrapure water (1 mg/ml), filtered through a 0.22‐mm filter, and injected into the GPC system. The mobile phase was ultrapure water, the flow rate was 0.3 ml/min, and the column temperature was 50°C.

#### 2.5.5 Evaluation of rheological behavior

The rheological properties of xanthan gum were determined using a rheometer (TA Instruments). The xanthan gum used to analyze the rheological properties was obtained at the completion of the fermentation (66 hr). The xanthan gum was dissolved in ultrapure water (10 mg/ml), and the rheological properties were determined in the shear rate range of 0.001–1,000/s at 25°C.

#### 2.5.6 Determination of xanthan gum whiteness

The whiteness of xanthan gum was determined using a fluorescence whiteness meter. After drying, crushing, and scraping using straightedge, samples of xanthan gum were placed in the measuring box, compacted, and analyzed.

## 3 RESULTS AND DISCUSSION

### 3.1 Identification of genes related to xanthomonadins in *Xanthomonas campestris* CGMCC15155

*Xanthomonas* produces xanthomonadins, which attach to the outer cell membranes, resulting in a yellow color. The biosynthesis of xanthomonadins involves a region of approximately 25 kbp that includes seven transcription units (*pigABCDEFG*). In this study, we knocked out the gene Xcc4015, which encodes an AMP‐ligase and locates at the *pigA* site. The wild‐type strain was yellow in color, while the *pigA* mutant was white, confirming that the *pigA* gene is indeed critical for the synthesis of xanthomonadins. To compare the amounts of xanthan gum produced by the *pigA* mutant and the wild type, fermentation was carried out in 500 ml Erlenmeyer flasks. The viscosity of the fermentation broth and xanthan gum yield of the *pigA* mutant (2509 mPa s and 25.7 g/L, respectively) were lower than those of the wild type (2799 mPa s and 28.9 g/L, respectively; Table 2). Thus, *Xanthomonas* lacking xanthomonadins showed decreased xanthan production, which was consistent with previous reports (Poplawsky & Chun, 1997; Poplawsky, Urban, & Chun, 2000). There may be a relationship between the synthesis of xanthomonadins and the level of extracellular polysaccharides.

**Table 2:** Xanthan gum yields and fermentation broth viscosities of the wild type, Δ*pigA*, and Δ*pigA*::*vgb* strains of *Xanthomonas campestris* CGMCC15155 in Erlenmeyer flasks

	Wild type	Δ*pigA*	Δ*pigA*::*vgb*
Yield (g/L)	28.9 ± 0.4	25.7 ± 0.3	30.8 ± 0. 7
Broth viscosity (mPa s)	2799 ± 47	2509 ± 25	2879 ± 45

All values are the means of three cultures replicated three times.

### 3.2 Confirmation of *pigA* gene inactivation and *vgb* gene insertion

The *vgb* gene encodes VHb. The heterologous expression of VHb not only allows the bacteria to grow in oxygen‐depleted environments but also increases cell growth and metabolite yield. The yield of *ΔpigA* was lower than that of the wild type; therefore, to obtain a strain whose yield was comparable to that of the wild‐type strain, engineered bacteria were reconstructed and the *vgb* gene (under the control of the LacZ promoter) was introduced into the wild‐type *X. campestris* CGMCC15155 *pigA* gene region to achieve simultaneous knockout of *pigA* (Figure 1).

The wild‐type colony appeared yellow, whereas the constructed mutant strain Δ*pigA*::*vgb* was white, confirming that xanthomonadin synthesis was successfully inactivated by *vgb* insertion (Figure 1b,c).

### 3.3 Determination of *vgb* gene expression

**Figure 2:**
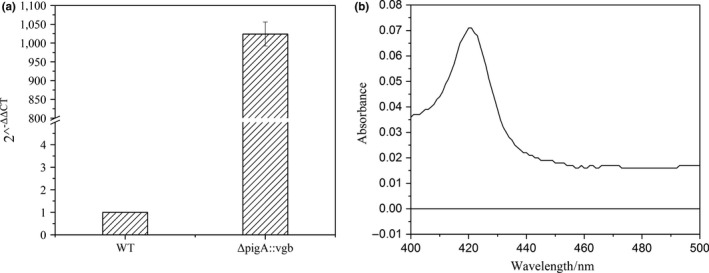
Confirmation of the expression of the *vgb* gene. (a) Confirmation of the *vgb* transcription by RT‐qPCR. The 16srDNA and *vgb* genes were quantified using primers 16sdl1/16sdl2 and *vgb*dl1/*vgb*dl2, respectively, and the expression level of *vgb* gene in WT strain was defined as 1. (b) CO differential chromatograms of the Δ*pigA*::*vgb* strain (upper) and wild type (lower), verifying the expression of VHb by *vgb* gene

The *vgb* gene in the Δ*pigA*::*vgb* strain was transcribed normally, while it was not detected in the wild‐type strain (Figure 2a). The combination of VHb and oxygen is similar to the combination of myoglobin and oxygen, and a CO–protein complex can be formed after the VHb protein is deoxidized, by introducing CO gas into the treated solution of wild‐type and Δ*pigA*::*vgb* cells. The characteristic absorption peak of the CO–protein complex appeared at approximately 420 nm, while the control strain did not produce any absorption peak (Choc, Webster, & Caughey, 1982). Thus, CO differential spectroscopy can be used to identify if VHb is active in an exogenous host. In this study, the CO differential chromatograms showed that Δ*pigA*::*vgb* had a significant absorption peak at 420 nm, whereas the wild type did not (Figure 2b). This verifies that the *vgb* gene in the Δ*pigA*::*vgb* strain expressed an active VHb protein.

### 3.4 Cell growth, xanthan gum concentration, and viscosity in batch cultivation

**Figure 3:**
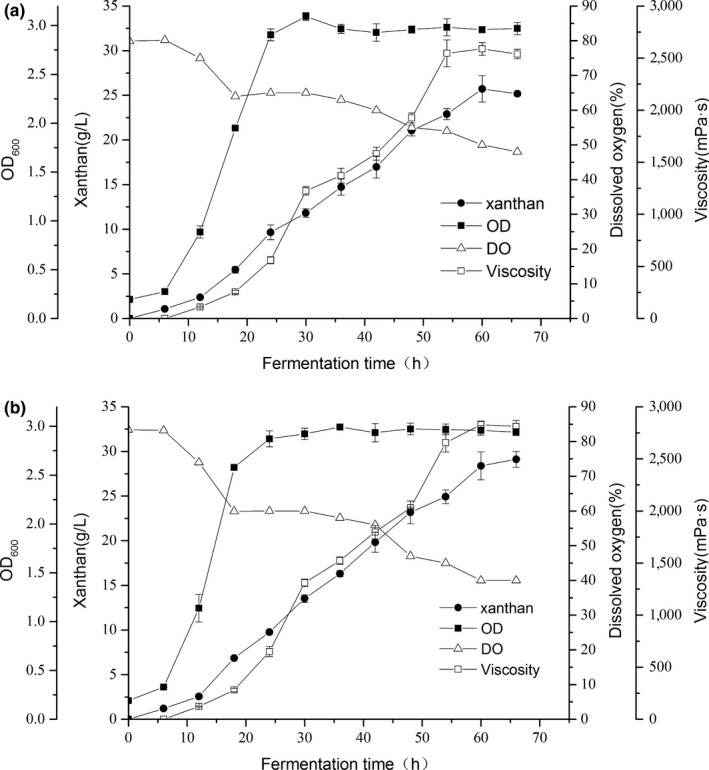
Batch fermentation profile of Δ*pigA* strain (a) and Δ*pigA*::*vgb* strain (b) in 5 L bioreactors for 66 hr. The cells were grown under the same fermentation conditions (error bars indicate *SD*)

To determine whether VHb has biological activity and how it affects xanthan production, the Δ*pigA*::*vgb* and Δ*pigA* strains were fermented in a bioreactor that can detect dissolved oxygen. The viscosity and the yield of xanthan gum of the Δ*pigA*::*vgb* were higher than that of Δ*pigA* during the whole fermentation process (Figure 3). After 66 hr of cultivation, the yield of Δ*pigA* was 25.2 ± 0.2 g/L and that of Δ*pigA*::*vgb* was 29.1 ± 0.4 g/L. Thus, the expression of *vgb* gene increased the yield of xanthan gum by 15.6%. Similarly, the viscosity of the fermentation broth of the strain expressing *vgb* was higher than that of the Δ*pigA* strain. The viscosity of the fermentation broth of the Δ*pigA* strain was 2539 ± 45 mPa s, while it was 2812 ± 57 mPa s in Δ*pigA*::*vgb*, representing an increase in the viscosity of the Δ*pigA*::*vgb* fermentation broth of 10.8%. The optical density (as a measure of the growth rate) of the two strains was analyzed. The results showed that the growth rate of Δ*pigA*::*vgb* was higher than of Δ*pigA*; however, at the end of the stationary stage, the biomass of the two strains was almost the same. The oxygen concentration in the fermentation broth was detected using a dissolved oxygen electrode. The dissolved oxygen in the fermentation broth of the two strains was basically the same at the initial stage of fermentation. As the viscosity of the Δ*pigA*::*vgb* fermentation broth became higher than that of Δ*pigA*, the dissolved oxygen of *ΔpigA::vgb* became gradually lower than that of Δ*pigA* (Figure 3). However, the growth rate of Δ*pigA*::*vgb* and the yield of xanthan gum were both higher than those of Δ*pigA* under conditions of lower dissolved oxygen concentration, indicating that VHb could effectively promote cell growth and the production of xanthan gum. Knockout of pigment‐related genes led to a decrease in xanthan gum yield; however, the expression of VHb increased xanthan gum production by the engineered strain to wild‐type levels (Table 2).

### 3.5 Determination of xanthan gum properties

**Figure 4:**
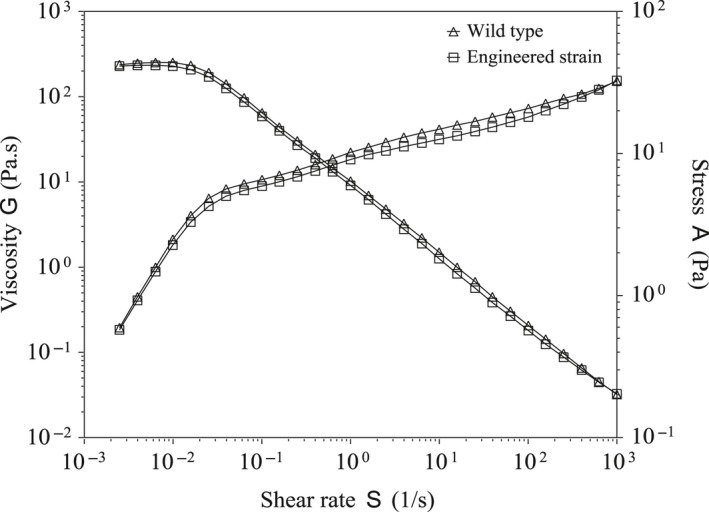
Rheological properties of xanthan gum produced by the engineered Δ*pigA*::*vgb* strain and the wild type. The concentration of xanthan gum was 1%, the detection temperature was 25°C, and the shear rate range was 0.001–1,000/s

Molecular weight is an important feature that affects the properties and applications of biopolymers (Ai et al., 2015). The molecular weights of the xanthan gum produced by the Δ*pigA*::*vgb* strain (6.8 × 10^6^ Da) and wild type (6.0 × 10^6^ Da) were essentially the same, as determined by GPC. The rheological properties of biopolymers, which reflect the microstructures and interactions between macromolecules and solvents in solution, are also important in industrial applications (Yu, Zhou, & Zhou, 2010). The relationships between the logarithm of the apparent viscosity of a 1% xanthan gum solution and the shear rate are shown for the Δ*pigA*::*vgb* strain and wild type in Figure 4. Both strains exhibited typical pseudoplastic rheology, characterized by decreasing apparent viscosity with increasing shear rate. The rheological properties of the xanthan gums from the engineered and wild‐type strains were basically the same. The level and change tendency of the apparent viscosity were same between the two kinds of xanthan gum (Figure 4). These results indicated that the engineered strain could replace the wild type in the industrial production of xanthan gum.

### 3.6 Extraction of xanthan gum

Residual xanthomonadins in xanthan gum cause the product to appear yellow. To obtain pure, white xanthan gum, the xanthomonadins are removed using excess ethanol, which increases the total cost of production. In the engineered Δ*pigA*::*vgb* strain, the production of xanthomonadins was almost nonexistent. Whiteness, reflecting the degree of white surface of the substance, is an important indicator in the food industry. As shown in Table 3, when the same volume of ethanol was used for xanthan gum extraction, the whiteness of the product produced by the Δ*pigA*::*vgb* strain was 65.20, while that of the xanthan gum produced by the wild type was 38.20 (equivalent to a 70.1% increase in whiteness). To obtain xanthan gum with the same whiteness, the Δ*pigA*::*vgb* product required ethanol at three times the volume of the fermentation broth, whereas the wild‐type xanthan gum required seven times the fermentation volume of ethanol (equivalent to a 133.3% decrease in the ethanol volume required by the engineered strain). These results indicated that the use of engineered bacteria in xanthan gum production greatly reduced the amount of ethanol required, which would decrease the extraction cost.

**Table 3:** Amount of ethanol used in xanthan gum extraction for the Δ*pigA*::*vgb* and wild‐type strains and corresponding whiteness values

Sample	Times of volume (ethanol/fermentation broth, v/v)	Whiteness
Δ*pigA*::*vgb*	3	65.20
Wild type	3	38.17
Wild type	4	42.16
Wild type	5	46.75
Wild type	6	55.89
Wild type	7	66.43

All values are means of three cultures replicated three times.

## 4 CONCLUSIONS

We engineered the xanthan gum‐producing strain of *X. campestris* CGMCC15155 and measured the yield and properties of the xanthan gum produced. The expression of *vgb* increased the growth rate and the metabolism of *X. campestris* CGMCC15155, which compensated for the decrease of xanthan gum yield caused by the absence of xanthomonadins, and allowed xanthan gum to be produced at wild‐type levels. The viscosity of the fermentation broth and the molecular weight and rheological properties of the xanthan gum were essentially the same for the Δ*pigA*::*vgb* and wild‐type strains. However, the engineered strain demonstrated the following advantages: When the same volume of ethanol was used, the whiteness of the engineered and wild‐type bacteria were 65.20 and 38.17, respectively. To extract xanthan gum with the same whiteness, three and seven times the fermentation volumes of ethanol were required for the engineered and wild‐type strains, respectively. Thus, the engineered strain reduced the volume of ethanol required for xanthan gum production by 133.3%. The results demonstrated that using the engineered bacteria in industrial xanthan gum production could greatly reduce the use of ethanol and the downstream production cost.

## CONFLICT OF INTEREST

All co‐authors have no conflicts of interest.
