# Supplementary material for: Construction and application of a Xanthomonas campestris CGMCC15155 strain that produces white xanthan gum
Source: Microbiologyopen. 2018 Apr 15;8(2):e00631. doi: 10.1002/mbo3.631 (PMC6391268; doi:10.1002/mbo3.631)
Supplement: Supplementary file 1 [file MBO3-8-e00631-s001.docx]

**Supplementary Table 1**

| Primers | | Sequence(5¢–3¢) | |  |
| --- | --- | --- | --- | --- |
| *pigA* -1SF | | GCTCTAGACGGTGACACCTTGCACAGAGAC | |  |
| *pigA* -1SR | | CAACCGCGTTTCACGATCGGCAGCTTCGACCCGGTATTC | |  |
| *pigA* -1XF | | GAATACCGGGTCGAAGCTGCCGATCGTGAAACGCGGTTG | |  |
| *pigA* -1SR | | CGAGCTCACCTCATCGCCCAGATACACC | |  |
| *pigA* -2SF | | GCTCTAGACGGTGACACCTTGCACAGAGAC | |  |
| *pigA* -2SR | | ACCCAGCTTTTGTTCCCTTTAGGCAGCTTCGACCCGGTATTC | |  |
| *pigA* -2XF | | GCTCAAGCGGTTGAATAACTCCGATCGTGAAACGCGGTTG | |  |
| *pigA* –2XR | | CGAGCTCACCTCATCGCCCAGATACACC | |  |
| P*vgb*-F | | GAATACCGGGTCGAAGCTGCCTAAAGGGAACAAAAGCTGGGT | |  |
| P*vgb*-R | | CAACCGCGTTTCACGATCGGAGTTATTCAACCGCTTGAGC | |  |
| *pigA* 1F | | CGGTGACACCTTGCACAGAGAC | |  |
| *pigA* 1R | | GCAGCTTCGACCCGGTATTC | |  |
| *pigA*2F | | GTCCCGCGCCTGCTGCT | |  |
| *pigA*2R | | GCTTGCATTGACGCTCATCC | |  |
| 16sdl1 | GCCCTCTGTCCCTACCATTGT | |  | |
| 16sdl2 | CTTGTCCTTAGTTGCCAGCAC | |  | |
| *vgb*dl1 | CTGTATTGAAGGAGCATGG | |  | |
| *vgb*dl2 | GCAAAATAGCTGGCAAATT | |  | |
